# Ensuring trust in COVID-19 data: A retrospective cohort study

**DOI:** 10.1097/MD.0000000000026972

**Published:** 2021-09-03

**Authors:** Daniel Antwi-Amoabeng, Bryce D. Beutler, Gurpreet Chahal, Sumaiya Mahboob, Nageshwara Gullapalli, Rudy Tedja, Farah Madhani-Lovely, Chris Rowan

**Affiliations:** a Department of Internal Medicine, University of Nevada Reno School of Medicine, Reno, NV; b Department of Pulmonary and Critical Care Medicine, Renown Health, Reno, NV; c Department of Infection Prevention, Renown Health, Reno, NV; d Institute for Heart and Vascular Health, Renown Regional Medical Center, Reno, NV.

**Keywords:** coronavirus, coronavirus disease, coronavirus disease-2019, epidemiology, health systems, severe acute respiratory syndrome coronavirus 2

## Abstract

There are no standardized methods for collecting and reporting coronavirus disease-2019 (COVID-19) data. We aimed to compare the proportion of patients admitted for COVID-19-related symptoms and those admitted for other reasons who incidentally tested positive for severe acute respiratory syndrome coronavirus 2 (SARS-CoV-2).

Retrospective cohort study

Data were sampled twice weekly between March 26 and June 6, 2020 from a “COVID-19 dashboard,” a system-wide administrative database that includes the number of hospitalized patients with a positive SARS-CoV-2 polymerase chain reaction test. Patient charts were subsequently reviewed and the principal reason for hospitalization abstracted.

Data collected during a statewide lockdown revealed that 92 hospitalized patients had positive SARS-CoV-2 test results. Among these individuals, 4.3% were hospitalized for reasons other than COVID-19-related symptoms but were incidentally found to be SARS-CoV-2-positive. After the lockdown was suspended, the total inpatient census of SARS-CoV-2-positive patients increased to 128, 20.3% of whom were hospitalized for non-COVID-19-related complaints.

In the absence of a statewide lockdown, there was a significant increase in the proportion of patients admitted for non-COVID-19-related complaints who were incidentally found to be SARS-CoV-2-positive. In order to ensure data integrity, coding should distinguish between patients with COVID-19-related symptoms and asymptomatic patients carrying the SARS-CoV-2 virus.

## 1 Introduction

Global prediction models indicate that a fourth wave of coronavirus disease-2019 (COVID-19), driven by the emerging delta variant, may be approaching. Accurate epidemiologic data is therefore of paramount importance in modeling resource utilization and health care systems planning. At present, there are no standardized methods for collecting and reporting COVID-19 data. In light of growing distrust in central governments to effectively manage this global crisis, data integrity has become all the more important to increase confidence in public health interventions.^[1]^ Indeed, even clinicians and public health experts have begun to question the quality of COVID-19 data that have been published in the medical literature^[2]^; recent study retractions have done little to alleviate these concerns.^[3]^

A central challenge of COVID-19 analytics is related to the high proportion of asymptomatic infections; morbidity and mortality data is inextricably linked to the number of incidental positive severe acute respiratory syndrome coronavirus 2 (SARS-CoV-2) tests among patients hospitalized for conditions unrelated to COVID-19. We aimed to investigate the distribution of patients who were admitted for COVID-19 symptoms versus those admitted for unrelated reasons who were incidentally found to have COVID-19. In addition, we compared data obtained during a statewide lockdown with that obtained in the postlockdown period to determine the effect of community spread on the rate of incidental positive tests.

## 2 Methods

Between March 26 and June 6, 2020, we sampled data from our “COVID-19 Dashboard,” a system-wide administrative database that includes the number of hospitalized patients with a positive SARS-CoV-2 polymerase chain reaction test. We defined “incidental positive cases” as patients hospitalized for non-COVID-related conditions who tested positive for SARS-CoV-2 but were represented as hospitalized COVID-19 patients in the database. Using the Mann–Whitney *U* test, we compared the mean daily frequency of incidental positive cases during and after a statewide lockdown, which was imposed between March 26 and April 30 and during which time all nonessential businesses were required to close and nonemergent surgical procedures were postponed. The difference in the proportion of incidental positive cases during and after the lockdown was assessed using a 2-sample Chi square test of proportions at 95% confidence. Stata version 16 (StataCorp., College Station, TX) was used for the statistical analysis.

The study was exempt from approval by the Institutional Review Board, as it was conducted as a quality improvement project.

## 3 Results

This study included 220 hospitalizations between March 26 and June 6, 2020. All patients were coded as being hospitalized for COVID-19. The total rate of incidental SARS-CoV-2 positivity in the cohort was 13.6%.

Data collected between March 26 and April 30 (33 days of lockdown) revealed that 92 hospitalized patients had positive SARS-CoV-2 test results. Among these individuals, 4.3% were hospitalized for reasons other than COVID-19 but were incidentally found to be SARS-CoV-2-positive. Between May 1 and June 6 (33 days of re-opening), the total inpatient census of SARS-CoV-2-positive patients increased to 128, 20.3% of whom were hospitalized for non-COVID-19-related complaints.

**Figure 1:**
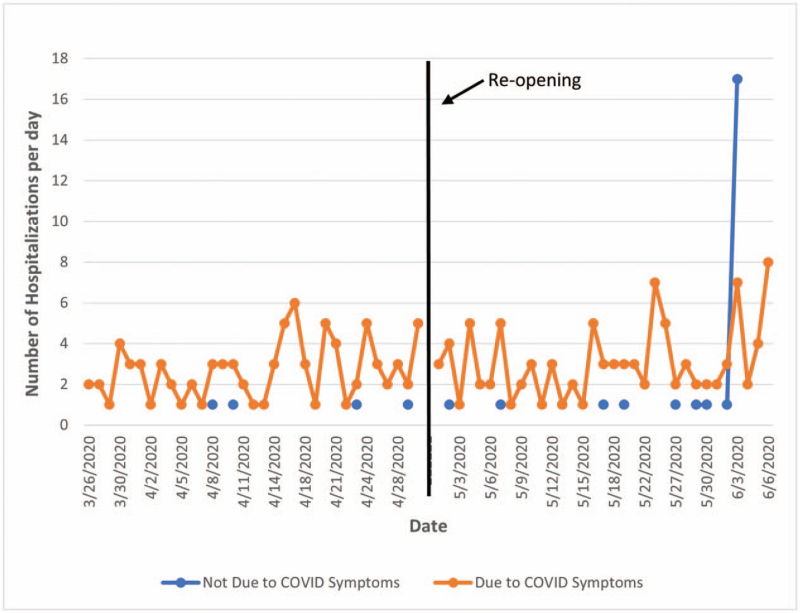
Trends in the daily number of hospitalizations of SARS-CoV-2 positive patients due to COVID symptoms versus not due to COVID symptoms. Vertical line (arrow) denotes the transition from the lockdown period (March 24^th^ to April 30^th^) and the re-opening period (May 1^st^ to June 6^th^). COVID = coronavirus disease, SARS-CoV-2 = severe acute respiratory syndrome coronavirus 2.

**Figure 2:**
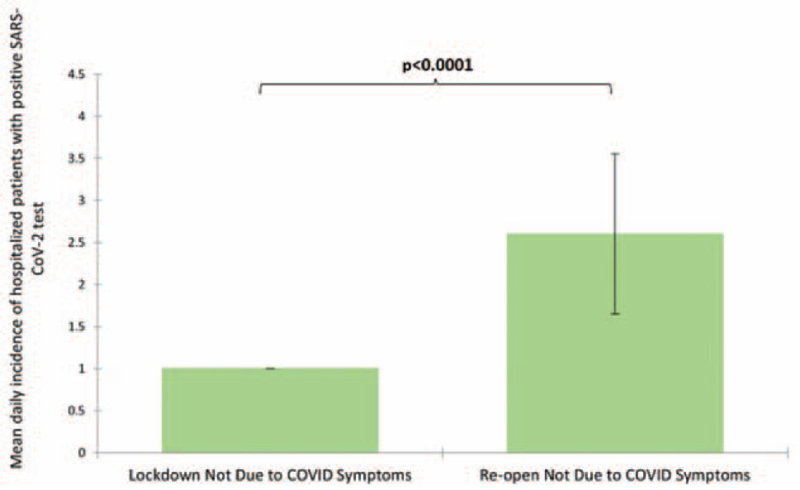
Comparison of the mean number of daily hospitalizations (error bars = confidence intervals) of patients who did not have COVID-19 symptoms during the lockdown period (March 24^th^ to April 30^th^) and the re-opening (May 1^st^ to June 6^th^). There were significantly more incidental SARS-CoV-2 hospitalization during the re-opening period (2.6 versus 1.0). COVID-19 = coronavirus disease-2019, SARS-CoV-2 = severe acute respiratory syndrome coronavirus 2.

The daily trend in SARS-CoV-2-positive patients remained stable in the lockdown period but increased approximately 2 weeks after the re-opening (Fig. 1). The mean number of daily non-COVID-19-related admissions was significantly higher after the re-opening period as compared to the lockdown period (2.6 versus 1, respectively; Fig. 2). In addition, there was a statistically significant (*P* *=* .001) increase in the proportion of incidental positive SARS-CoV-2 tests between the lockdown and immediate postlockdown periods (4.3% and 20.3%, respectively). The relationship between timeline and hospitalization type is further illustrated in Table 1.

**Table 1:** Cross-tabulation of COVID-19 hospitalization type with respect to the lockdown timeline. There was an association between the timeline and hospitalization type (*P*-value for χ2 test of association <.001).

	Type of COVID-19 hospitalization		
Timeline	Incidental	Symptomatic	Row total
Lockdown	4 (4.3%)	88 (95.7%)	92 (100%)
Re-opening	26 (20.3%)	102 (79.7%)	128 (100%)
Row total	30 (13.6%)	190 (86.4%)	220 (100%)

COVID-19 = coronavirus disease-2019.

## 4 Discussion

In June 2020, the National Institutes of Health announced the creation of the National COVID Cohort Collaborative, which aims to collect and study medical record data on persons diagnosed with COVID-19.^[4]^ Although the National COVID Cohort Collaborative is a laudable undertaking, the quality of the collected data will determine the utility of the database as well as the external validity of findings from studies using the database. We have concerns about the variability in data collection across institutions and are wary of coding practices that do not distinguish asymptomatic patients from those hospitalized with COVID-related symptoms. Here, we describe our experience from our hospital in northern Nevada.

We investigated incidence patterns of COVID-19 among patients in our 808-bed tertiary referral center to assess the distribution of patients who were admitted *for* COVID-19 versus those *with* an incidental finding of COVID-19. We hypothesized that universal testing of patients presenting to the emergency department would result in a higher number of positive cases among hospitalized patients. We further hypothesized that the proportion of patients admitted with a diagnosis of COVID-19 – with or without COVID-19-related symptoms – would increase in tandem with the prevalence of the disease in the community.

The increase in the number of hospitalized patients with positive SARS-CoV-2 tests results can likely be attributed to rising community prevalence in the setting of increased admissions for non-COVID-19 complaints during the postlockdown period; data show that emergency department visits were markedly decreased during state and national lockdowns, likely due to patient concerns for COVID-19 exposure.^[5]^ During the lockdown and immediate postlockdown periods, our hospital instituted a policy of SARS-CoV-2 screening for all patients who were to be hospitalized. A consequence of this policy was an increase in the number of positive cases among hospitalized patients. Examples include a teenager with a traumatic femur fracture, a primigravid patient in labor, and a middle-aged man with appendicitis, all of whom were incidentally found to be SARS-CoV-2-positive but were erroneously represented as hospitalized COVID-19 patients in the hospital administrative database.

Two new provisional International Classification of Diseases, Tenth Edition (ICD-10) codes are used for establishing a diagnosis of COVID-19: U07.1, which denotes positive identification of the SARS-CoV-2 virus and U07.2, which indicates no laboratory confirmation of the virus in the setting of a clinical diagnosis of COVID-19. These codes are utilized for both clinical and epidemiological coding of the disease and will be used to monitor incidence, prevalence, hospitalizations, and in-hospital outcomes.^[6]^ Since up to 30% of patients who test positive for SARS-CoV-2 may be asymptomatic, hospital data standardization is vital to clearly distinguish incidental from symptomatic cases. Indeed, there is a need to define these codes to indicate which patients suffered significant clinical effects of COVID-19, as the current recommendation is to use additional diagnoses to identify disease manifestations.^[7]^ It will also be prudent to define ICD-10 codes for organ-specific COVID-19 dysfunction in affected patients.

Notably, data suggest that a significant proportion of COVID-19 cases go unreported^[8,9]^; this affects calculation of the R_0_ as well as the case fatality rate. Estimates of the true disease burden rely on correlation of symptomatic, asymptomatic, and unreported cases, and thus it is essential to define ICD-10 codes appropriately to distinguish between incidental and nonincidental cases.

This report draws attention to inherent flaws in the current practice of collecting administrative data on COVID-19 hospitalizations. We believe the practices described herein are representative of the general United States hospital setting. However, we are limited by our inability to include data from other hospitals in our analysis. Further, our sample size is rather small and may underestimate the proportion of hospitalizations with SARS-CoV-2 infection without clinical evidence of COVID-19 disease.

## 5 Conclusion

Scientific evidence represents the foundation of all effective public health policy. Implementation of population-level interventions often require years of epidemiologic data collection. The COVID-19 pandemic is rapidly evolving as new variants emerge, and the medical community is frantically trying to stem the spread of the disease while collecting data to inform public health interventions. However, ensuring data integrity is crucial. We must establish clear definitions of disease states and associated outcomes in order to safeguard clinical and epidemiological research; we believe that this will ultimately strengthen interventions to control the disease. We need to change reporting to designate which patients are admitted *for* COVID-19 and which are admitted *with* COVID-19. Furthermore, designating the extent of organ involvement will be valuable in monitoring long-term sequalae of COVID-19. Public trust must be regained in our collective efforts to manage this pandemic.

## Author contributions

**Conceptualization:** Daniel Antwi-Amoabeng, Gurpreet Chahal, Nageshwara Gullapalli.

**Data curation:** Daniel Antwi-Amoabeng, Sumaiya Mahboob, Rudy Tedja.

**Funding acquisition:** Gurpreet Chahal.

**Investigation:** Daniel Antwi-Amoabeng, Bryce David Beutler, Rudy Tedja, Farah Madhani-Lovely.

**Methodology:** Rudy Tedja.

**Supervision:** Chris Rowan.

**Writing – original draft:** Bryce David Beutler.

**Writing – review & editing:** Nageshwara Gullapalli.
